# Retroviral integration site selection: a running *Gag*?

**DOI:** 10.15698/mic2018.12.663

**Published:** 2018-11-19

**Authors:** Paul Lesbats, Vincent Parissi

**Affiliations:** 1 Fundamental Microbiology and Pathogenicity Laboratory, UMR 5234 CNRS, SFR TransBioMed. Bordeaux, France.; 2 International Associated Laboratory (LIA) of Microbiology and Immunology, CNRS / Heinrich Pette Institute-Leibniz Institute for Experimental Virology, Bordeaux, France.; 3 Viral DNA Integration and Chromatin Dynamics Network (DyNAVir), Bordeaux, France.

**Keywords:** retrovirus, integration, nucleosome, chromatin-binding, Gag

## Abstract

The ability of retroviruses to integrate their genomes into host chromatin is a key step for the completion of their replication cycle. Selection of a suitable chromosomal integration site has been described as a hierarchical mechanism involving both cellular and viral proteins but the exact molecular determinants are still unclear. We recently showed that the spumaretrovirus prototype foamy virus (PFV) Gag protein is acting as a chromatin tether by interacting with the nucleosome acidic patch (Lesbats et al. *PNAS* 114(21)). Disruption of the nucleosome binding leads to a dramatic delocalization of both the viral particles and the integration sites accompanied with a reduction of integrated genes expression. These data show for the first time a direct interaction between retroviral structural proteins with the host chromosomes, and highlight their importance in the integration sites selection.

The retroviral Gag proteins encoded by the *gag* (group antigen) gene compose the main structural components of the viral particle, with the exception of the envelope. Their involvement during retroviral cycle is pleiotropic, ranging from intracellular viral trafficking to viral assembly and integration. Retroviral integration site selection of a suitable chromatin environment is crucial for the fate of both the virus and the cell. Several studies on the lentivirus Human Immunodeficiency Virus-1 (HIV-1) and gammaretrovirus Murine Leukemia Virus (MLV) showed that their integration site selection is partly dictated by the interaction of their integrases (IN) with host cell chromatin-bound proteins, like LEDGF/p75 and BRD2-4, respectively. The chromatin-bound host factors act as tethers or receptors for the cognate pre-integration complexes. In addition, an increasing body of recent evidence demonstrates that the HIV-1 capsid protein, a Gag product, is involved in integration site selection via binding to the cellular cleavage and polyadenylation specificity factor 6 (CPSF6) protein. This interaction induces a directing of the bulk of integration into gene dense regions of the nucleus. For the Spumaretrovirus family, previous data showed a tight association of the Gag proteins with mitotic chromosomes. The functional significance as well as the molecular determinants of this interaction were unclear. By using biochemistry and x-ray crystallography we could obtain the high-resolution structure of the chromatin-binding sequence (CBS) domain of the PFV Gag protein bound to a nucleosome. The CBS peptide adopts an elongated conformation along the face of the nucleosome core. The CBS-nucleosome complex involves one H2A-H2B heterodimer and both H3 histones chains with an array of hydrogen bounds and hydrophobic contacts between the two partners. We noted that PFV Gag Arg540 projects into the H2A–H2B acidic patch to interact with H2A carboxylates Glu61, Asp90, and Glu92. Interestingly, most of the chromatin factors-nucleosomes structures solved to date show the use of a conserved arginine anchor residue for the interaction of the H2A-H2B acidic patch carboxylates. Consistently, substitution of Gag Arg540 as well as conserved Tyr537 and Leu539 abolish *in vitro* interaction with the nucleosome. In agreement with published results, immunofluorescence of incoming viral particles showed a colocalization with centrosomes in interphase cells and a chromatin association during mitosis. The R540Q mutant viruses still localize around centrosomes during interphase, but lost the capacity to associate with mitotic chromosomes, phenocopying the *in vitro* substitutions results. Quantification of viral infectivity in the context of viruses harboring the R540Q substitution showed a modest two-fold defect compared to wild type (WT) viruses. Interestingly, the integration step was found unaffected suggesting an expression alteration of the integrated genes. To get deeper insights on the role of the Gag-chromatin interaction, we mapped the integration sites of WT and R540Q PFV in various cell types. In agreement with previously published results and in contrast to HIV-1 and MLV, PFV avoids integration into genes. Surprisingly, the virus showed varied preference for heterochromatic or promoters regions depending on the cell line used for infection (with HT1080 and HepG2 showing the most divergences). Indeed, while heterochromatin regions like constitutive lamina associated domains (cLADs) or giemsa positive cytobands are favored in HT1080 cells, they are disfavored in HepG2. Conversely, compared to HT1080, the virus integrates more often near transcription start sites and CpG islands in HepG2 cells. Nevertheless, analysis of integration sites of the R540Q virus revealed a massive redistribution of integration toward centromeres in all cell lines studied. These data demonstrate that Gag tethering to host chromatin is critical for the virus to select an optimal integration site.

In light of the discordances observed in integration sites selection of the wt virus in the different cell lines, it is possible that the conditions of infections can widely affect the outcome of the integration targeting observed. Indeed, in contrast to HepG2, HT1080 cells are highly permissive to foamy virus (FV) infection. Since deep sequencing analyses require many unique integration sites, infection experiments are performed under high multiplicity of infection (MOI). We can then speculate that in HT1080 cells, a massive amount of viral particles can enter each cell, possibly titrating specific cellular proteins or pathways that will impact the resulting integration site selection. In HepG2 such “viral flooding” will be limited, potentially revealing the original FV integration preference. This hypothesis would require more analyses, specifically under natural zoonotic infections.

In contrast to foamy viruses, orthoretroviral Gag proteins are matured into separate polypeptides. Recently, it has been described that gammaretrovirus MLV Gag product p12 associates directly to host nucleosomes. Whether this p12 protein is involved in gammaretroviral integration site selection is currently unclear, but the correct tethering to chromatin is critical, since abolishment of p12-nucleosome binding abrogates viral infectivity.

The role of Gag in chromatin targeting in the other retroviral family is currently obscure. Lentiviruses rely on the capsid-binding partners like CPSF6 or nucleoporin NUP153 to gain access to gene dense chromosomal regions via an active nuclear import process. In the case of spumaretrovirus and gammaretrovirus this function is achieved by direct binding of Gag products to host chromatin during mitosis.

Chromatin binding by viral proteins is not restricted to retroviruses as it was also described for Kaposi’s sarcoma herpes virus (KSHV) latency-associated nuclear antigen (LANA) and cytomegalovirus (CMV) immediate early 1 protein (IE1). As described for PFV Gag, structural analyses showed a conserved interaction mode using an arginine anchor motif poking to the acidic patch. Interestingly, biophysical approaches revealed an interesting feature about these acidic patch binders viral proteins. As the acidic patch constitutes the binding pocket for the neighboring histone H4 tail during higher order chromatin assembly, it was shown that competition for the acidic patch using the viral proteins LANA and IE1 could induce an alteration of the chromatin compaction *in vitro*. Whether this feature is also expressed during viral replication is currently not known. As foamy viruses favor integration into deep heterochromatic regions of the chromosomes, it is tempting to speculate that the binding of Gag to compacted nucleosomes might locally remodel the chromatin architecture in order to create a replication/gene-expression permissive environment (Figure 1). Such a hypothesis is currently under investigation.

**Figure 1:**
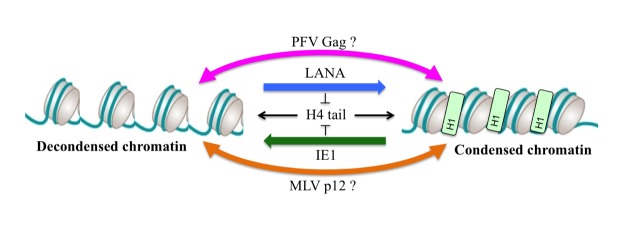
FIGURE 1: Hypothesis model for viral protein mediated chromatin remodeling. The ground state chromatin structure is mediated by H4 tail binding to the acidic patch. Alteration of the chromatin state can be achieved by competition of viral proteins with H4 tail for binding to the acidic patch leading to a viral replication-permissive chromatin structure. Abbreviations: H1, histone H1. H4, histone H4. See text for details.

Virus-host interaction is an arms race, which has shaped the biology we know today during the course of evolution. The retrovirus family originated more than 500 millions years ago and nowadays infects a broad range of hosts. From the oldest Foamy viruses to the most recent pandemic causative HIV-1, latest researches showed a new mechanism of regulation of integration site selection by the viral capsid/Gag. Further experiments investigating the potential involvement of Gag products from other retroviral genera on the targeting of integration is of great interest and will shed light on the replicative strategy evolved by retroviruses.

